# A retrospective clinical audit of general practices in Australia to determine the motivation for switch to dolutegravir/abacavir/lamivudine and clinical outcomes

**DOI:** 10.1177/0956462417730474

**Published:** 2017-09-13

**Authors:** Pedro E Ferrer, Mark Bloch, Norman Roth, Robert Finlayson, David Baker, Ken Koh, David Orth, Rimgaile Urbaityte, Dannae Brown, Fraser Drummond

**Affiliations:** 1 ViiV Healthcare, Research Triangle Park, NC, USA; 2 Holdsworth House Medical Practice, NSW, Sydney, Australia; 3 Prahran Market Clinic, Melbourne, VIC, Australia; 4 Taylor Square Private Clinic, Sydney, NSW, Australia; 5 East Sydney Doctors, Sydney, NSW, Australia; 6 Holdsworth House Medical Brisbane, Brisbane, QLD, Australia; 7 Gladstone Road Medical Centre, Brisbane, QLD, Australia; 8 GlaxoSmithKline, Uxbridge, UK; 9 ViiV Healthcare, Sydney, NSW, Australia

**Keywords:** Dolutegravir, HIV, switch, real world

## Abstract

The most common reasons for switching HIV-1 therapy in patients with virologic suppression are treatment regimen simplification and resolving tolerability issues. Single-pill regimens that include an integrase inhibitor are recommended options. A retrospective clinical audit was performed to determine the motivations for switching to dolutegravir (DTG)/abacavir (ABC)/lamivudine (3TC) at high HIV-caseload general practice clinics in Australia. The most common reasons for switching from a prior suppressive therapy to DTG/ABC/3TC were simplification of regimen, resolving toxicity/intolerance and patient preference (73%, 13% and 12%, respectively). Kaplan–Meier analysis showed that the probability of patients remaining on DTG/ABC/3TC therapy at 12 months was 95.1%. Switching to DTG/ABC/3TC from a range of other regimens was associated with a discontinuation rate of 3.2%, with 2.5% of patients discontinuing due to adverse events and no patients discontinuing due to virologic failure. Switching to DTG/ABC/3TC was a viable treatment strategy in this cohort of Australian patients.

## Introduction

The need to switch HIV-1 therapy because of virologic failure and drug resistance has decreased with improvements in antiretroviral therapy (ART). As a result of these improvements, there is now a rationale for switching therapy in some patients with virologic suppression. Reasons to consider switching therapy in such patients include adverse events (AEs) and simplification of regimen.^1–3^ Data on the outcomes of switching therapy are still evolving, therefore studies that provide insights into outcomes of patients post-switching therapy are important. The STRIIVING study demonstrated the non-inferiority of switching from a variety of antiretrovirals (ARVs) onto dolutegravir (DTG)/abacavir (ABC)/lamivudine (3TC) in comparison to staying on baseline (current) ART.^4^ The rate of AEs leading to discontinuation of DTG/ABC/3TC (4%) in this patient population was similar to that observed in DTG treatment-naive studies.^4–9^

The present paper describes a retrospective clinical audit at high HIV-caseload primary care practices in Australia to determine why virologically suppressed patients switched therapy to DTG/ABC/3TC fixed-dose combination and the clinical outcomes following this switch.

## Methods

Patients identified across six primary care practices, who had received DTG/ABC/3TC alone for ≥1 day following a switch from suppressive ART (<50 HIV-1 RNA copies/ml), were included. These patients were required to have switched to DTG/ABC/3TC between 1 April 2015 and 31 March 2016, and to have been maintained on DTG/ABC/3TC alone after the switch. Patient files were reviewed by each general practice and individual cases submitted via a systematic electronic survey.

The primary outcome was the percentage of patients remaining on DTG/ABC/3TC therapy with HIV-1 RNA < 50 copies/ml. Time on treatment was calculated from the date of first DTG/ABC/3TC script and censored on 1 April 2016. Kaplan–Meier survival methods were used to determine probability of DTG/ABC/3TC continuation at 12 months of treatment. All other results are descriptive.

This project was approved by an Australian private not-for-profit ethics committee registered with the National Health and Medical Research Council and all patient data were de-identified.

## Results

Data from 443 patients were included: 97% male and 45% ≥50 years. Of the 443 patients, two patients discontinued from DTG/ABC/3TC after 1 April 2016 and the data of one patient were received after the study closing date; the data from these three patients were included in the study. A summary of the most recent regimen prior to switching therapy is shown in Table 1.

**Table 1.** Summary of ART history and most recent regimen prior to switch (N = 443).

ART history		n (%)
Historical ARV resistance	Any	29 (6.5)
	None	414 (93.5)
Time on ART	<5 years	124 (28.0)
	5–10 years	119 (26.9)
	>10 years	200 (45.1)
Number of previous ART regimens	≥5	88 (19.9)
	3–4	125 (28.2)
	1–2	230 (52.0)
Most recent ART regimen		n (%)
Time on prior therapy	<2 years	242 (54.6)
	2–5 years	81 (18.3)
	>5 years	120 (27.1)
Single-pill regimen	–	50 (11.2)
Prior two NRTI^a^^,^^b^	ABC/3TC	300 (67.7)
	TDF/FTC	119 (26.9)
	AZT/3TC	5 (1.1)
	NRTI sparing	5 (1.1)
Prior core agent^a^^,^^c^	INSTI	258 (58.2)
	NNRTI	118 (26.6)
	PI	91 (20.5)
	EI	2 (0.5)
	MI	1 (0.2)
Prior DTG-basedregimen^a^	DTG + ABC/3TC (alone)	165 (37.2)
	Non DTG-based regimen	242 (54.6)

ABC/3TC: abacavir/lamivudine; ART: antiretroviral therapy; ARV: antiretroviral; AZT/3TC: zidovudine/lamivudine; DTG: dolutegravir; EI: entry inhibitor; INSTI: integrase strand transfer inhibitor; MI: maturation inhibitor; NNRTI: non-nucleoside reverse transcriptase inhibitor; NRTI: nucleoside reverse transcriptase inhibitor; PI: protease inhibitor; TDF/FTC: tenofovir/emtricitabine.

^a^Most recent regimen before DTG/ABC/3TC.

^b^Includes patients on single-pill regimens containing the combination NRTI; 14 patients had alternative combination NRTIs or a single NRTI in their regimen.

^c^Some patients were on multiple core agents.

**Figure 1.**
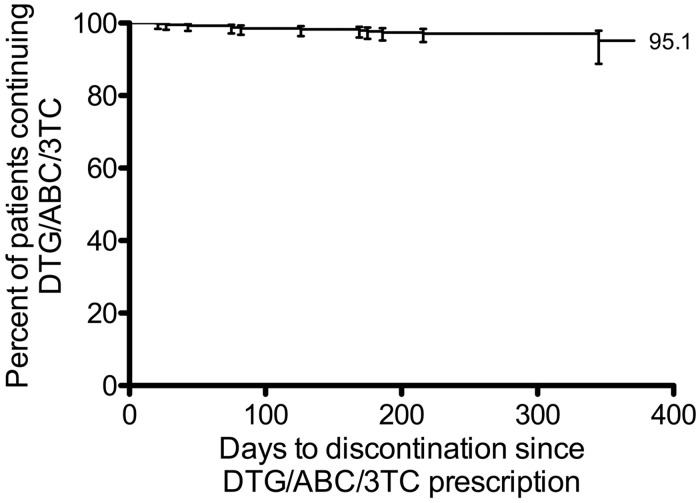
Per cent of patients remaining on DTG/ABC/3TC therapy after switching, based on pre-specified censorship on 31 March 2016 (N = 443).

The probability of patients remaining on DTG/ABC/3TC therapy at 12 months was 95.1% (95% CI 88.8–97.9) by Kaplan–Meier estimate (Figure 1).

The most common reasons patients switched therapy to DTG/ABC/3TC were simplification, toxicity/intolerance and patient preference (73%, 13% and 12%, respectively). After excluding prior DTG-based regimens as the most recent before the switch, toxicity/intolerance as a reason for switching therapy increased to 21%. Few (2%) patients switched therapy for reasons attributed to cost, suboptimal adherence or drug–drug interactions. The most common toxicities/intolerances leading to the discontinuation of prior therapy and switching to DTG/ABC/3TC were nervous system disorders, renal and urinary disorders, and gastrointestinal disorders. Pre-existing toxicity/intolerance events resolved in 43 of 58 (74%) patients, including nine of 13 (69%) with nervous system disorders, eight of 12 (67%) with renal and urinary disorders and ten of ten (100%) with gastrointestinal disorders. Patients with toxicity/intolerance events that did not resolve after switch onto DTG/ABC/3TC had a median time on treatment that was lower (191 days [IQR 100–239.5]) than that of the overall population (266 days [IQR 176–325]).

Fourteen patients (3.2%) discontinued DTG/ABC/3TC; none of these were due to virologic failure (Table 2). Discontinuations were mainly related to AEs (2.5%);  < 1% of patients discontinued due to a psychiatric event. The discontinuation rates for all subgroups were similar (0–5.6%), i.e.

**Table 2.** Patients who discontinued DTG/ABC/3TC.

Prior regimen^a^	Reason for switch to DTG/ABC/3TC	Days on DTG/ABC/3TC	Reason for DTG/ABC/3TC discontinuation^b^	Event	Subsequent regimen	Returned to prior regimen	AE ceased	Regained/ maintained viral suppression
DTG + ABC/3TC	Simplification	175	Toxicity/intolerance	Headache^c^	DTG + ABC/3TC	Yes	Yes	Yes
ATV/r + ABC/3TC	Simplification	75	Blip	Single event of 63 copies/ml	ATV/r + ABC/3TC	Yes		Yes
DTG + ABC/3TC	Simplification	381	Toxicity/intolerance	Creatinine	EVG/c/TDF/FTC	No	No	Yes
ATV/r + TDF/FTC	Simplification	82	Toxicity/intolerance	Abdominal discomfort	ATV/r + TDF/FTC	Yes	Yes	Yes
RAL + ABC/3TC	Simplification	126	Toxicity/intolerance	Insomnia	RAL + ABC/3TC	Yes	Yes	Yes
NVP + TDF/FTC^d^	Toxicity/intolerance	75	Toxicity/intolerance	Anxiety^e^	NVP + TDF/FTC	Yes	Yes	Yes
RAL + ABC/3TC^d^	Toxicity/intolerance	169	Toxicity/intolerance	Dry mouth	RAL + ABC/3TC	Yes	Yes	Yes
RAL + TDF/FTC	Simplification	27	Toxicity/intolerance	Rash	RAL + TDF/FTC	Yes	Yes	Yes
DTG + ABC/3TC	Simplification	43	Drug–drug interactions	Cigarettes and risk of CVD	ATV/r + TDF/FTC	No		Yes
DTG + ABC/3TC^f^	Patient preference	304	Toxicity/intolerance	Malaise^g^	NVP + ABC/3TC	No	Yes	Yes
NVP + ABC/3TC	Simplification	199	Patient preference		NVP + ABC/3TC	Yes		Yes
DTG + ABC/3TC	Simplification	345	Toxicity/intolerance	Anxiety	DRV/r + ABC/3TC	No	Yes	Yes
RAL + DRV/r + ABC/3TC	Simplification	186	Toxicity/intolerance	Rhabdomyolysis^h^	RPV/TDF/FTC	No	Yes	Yes
RAL + NVP + 3TC	Simplification	21	Toxicity/intolerance	Nausea	RAL + NVP + 3TC	Yes	Yes	Yes

3TC: lamivudine; ABC: abacavir; AE: adverse event; ATV/r: atazanavir/ritonavir; c: cobicistat; CVD: cardiovascular disease; DRV/r: darunavir/ritonavir; DTG: dolutegravir; EVG: elvitegravir; NVP: nevirapine; RAL: raltegravir; RPV: rilpivirine; TDF/FTC: tenofovir/emtricitabine.

^a^Switched from prior regimens for simplification, with three exceptions.

^b^Median time on DTG/ABC/3TC treatment was 147.5 (IQR 75–195.75) days in these patients.

^c^This subject was entered two days after study closure.

^d^Discontinued prior regimen due to a pre-existing toxicity/intolerance.

^e^History of anxiety.

^f^Discontinued prior regimen due to patient preference.

^g^Aches, shortness of breath, tight chest, coughing, fatigue.

^h^Serious AE: onset bilateral arms, required hospitalisation.

- history of ARV resistance (any, 3.4%; none, 3.1%)
- number of previous regimens (≥5, 1.1%; 3–4, 5.6%; 1–2, 2.6%)
- time on most recent regimen (<2 years, 3.3%; 2–5 years, 3.7%; 5 years, 2.5%)
- most recent agent class in prior regimen (integrase strand transfer inhibitor, 3.9%; two nucleoside reverse transcriptase inhibitors [2NRTIs; TDF/FTC-ABC/3TC], 2.5–3.3%; non-NRTI, 2.5%; protease inhibitor, 3.2%)
- rationale for regimen switch (simplification, 3.1%; toxicity/intolerance, 3.5%; patient preference, 2.0%)
- DTG not used in prior regimen (3.7%) or a combination of DTG + ABC/3TC alone not used in prior regimen (3.0%)

Nine of 14 patients who discontinued DTG/ABC/3TC switched therapy back to their prior regimen (Table 2).

## Discussion

This retrospective clinical audit demonstrated that the relative probability of staying on DTG/ABC/3TC therapy at 12 months after switching from another regimen was 95.1%.

The most common reason for switching from a prior suppressive therapy to DTG/ABC/3TC was simplification. For those who switched therapy due to toxicity/intolerance, most pre-existing events, including over two-thirds of nervous system disorders, resolved. Of patients who ceased DTG/ABC/3TC therapy, all remained virologically suppressed on their subsequent regimen, which was often the regimen they had switched from.

The rate of discontinuations due to AEs in this study was low and comparable to that of the phase 3b stable switch study STRIIVING (2.5% and 4.0%, respectively).^4^ Other randomised controlled trials assessing stable switch strategies to single-pill regimens containing rilpivirine or elvitegravir have reported similar discontinuation rates due to AEs.^10^^–14^ The rates of discontinuations due to psychiatric AEs observed in this Australian cohort were consistent with the phase 3 clinical trials of DTG in treatment-naïve patients^5^^–9^ as well as many real-world evidence studies.^15^^–19^ Some real-world evidence studies have reported higher rates of discontinuations due to psychiatric AEs,^20^^–22^ possibly because of differences in study populations, or perhaps due to reporting and/or channelling bias. This tenet is supported, in part, by an analysis of the OPERA cohort study, in which patients receiving DTG were found to be more likely to have a history of psychiatric disorder at baseline than patients receiving other core agents.^19^

This audit report was subject to the general limitations of a retrospective cohort analysis, including limits to data collection imposed by pre-specified end points in the study protocol. Consequently, data were not collected for a range of parameters including other regimens switched to during similar time frames, AEs that did not lead to discontinuation and the severity of AEs leading to discontinuation. In addition, there are limitations associated with use of DTG/ABC/3TC, including the need to test for HLA-B*5701 allele status before initiating therapy and, since DTG/ABC/3TC is a fixed dose tablet, it should not be prescribed for patients requiring dose adjustment.

In this real-world retrospective audit, switching to DTG/ABC/3TC from a range of other regimens showed a low rate of discontinuation. Few patients were reported to discontinue due to AEs and no patients discontinued due to virologic failure, supporting DTG/ABC/3TC as a viable treatment strategy in this Australian patient population.
